# Phosphorylation of slit diaphragm proteins NEPHRIN and NEPH1 upon binding of HGF promotes podocyte repair

**DOI:** 10.1016/j.jbc.2021.101079

**Published:** 2021-08-13

**Authors:** Ashish K. Solanki, Ehtesham Arif, Pankaj Srivastava, Christopher M. Furcht, Bushra Rahman, Pei Wen, Avinash Singh, Lawrence B. Holzman, Wayne R. Fitzgibbon, Milos N. Budisavljevic, Glenn P. Lobo, Sang-Ho Kwon, Zhe Han, Matthew J. Lazzara, Joshua H. Lipschutz, Deepak Nihalani

**Affiliations:** 1 Department of Medicine, Medical University of South Carolina, Charleston, South Carolina, USA; 2 Department of Biochemistry and Molecular Genetics, University of Colorado Anschutz Medical Campus, Aurora, Colorado, USA; 3 Department of Chemical and Biomolecular Engineering, University of Pennsylvania, Philadelphia, Pennsylvania, USA; 4 Department of Medicine, University of Maryland School of Medicine, Baltimore, Maryland, USA; 5 Department of Medicine, University of Pennsylvania, Philadelphia, Pennsylvania, USA; 6 Department of Cellular Biology and Anatomy, Augusta University, Augusta, Georgia, USA; 7 Department of Chemical Engineering, University of Virginia, Charlottesville, Virginia, USA; 8 Department of Medicine, Ralph H. Johnson Veterans Affairs Medical Center, Charleston, South Carolina, USA; 9 Division of Kidney, Urologic and Hematologic Diseases, National Institutes of Health, Bethesda, Maryland, USA

**Keywords:** HGF, phosphorylation–dephosphorylation, SHP-2, podocytes, CD, cytoplasmic domain, ECD, extracellular domain, FL, full length, GST, glutathione S-transferase, HGF, hepatocyte growth factor, KD, knockdown, MET, mesenchymal epithelial transition, PS, protamine sulfate, SHP-2, SH2 domain–containing protein tyrosine phosphatase-2, SNS, sticks-and-stones

## Abstract

Phosphorylation (activation) and dephosphorylation (deactivation) of the slit diaphragm proteins NEPHRIN and NEPH1 are critical for maintaining the kidney epithelial podocyte actin cytoskeleton and, therefore, proper glomerular filtration. However, the mechanisms underlying these events remain largely unknown. Here we show that NEPHRIN and NEPH1 are novel receptor proteins for hepatocyte growth factor (HGF) and can be phosphorylated independently of the mesenchymal epithelial transition receptor in a ligand-dependent fashion through engagement of their extracellular domains by HGF. Furthermore, we demonstrate SH2 domain–containing protein tyrosine phosphatase-2–dependent dephosphorylation of these proteins. To establish HGF as a ligand, purified baculovirus-expressed NEPHRIN and NEPH1 recombinant proteins were used in surface plasma resonance binding experiments. We report high-affinity interactions of NEPHRIN and NEPH1 with HGF, although NEPHRIN binding was 20-fold higher than that of NEPH1. In addition, using molecular modeling we constructed peptides that were used to map specific HGF-binding regions in the extracellular domains of NEPHRIN and NEPH1. Finally, using an *in vitro* model of cultured podocytes and an *ex vivo* model of *Drosophila* nephrocytes, as well as chemically induced injury models, we demonstrated that HGF-induced phosphorylation of NEPHRIN and NEPH1 is centrally involved in podocyte repair. Taken together, this is the first study demonstrating a receptor-based function for NEPHRIN and NEPH1. This has important biological and clinical implications for the repair of injured podocytes and the maintenance of podocyte integrity.

With increasing knowledge of podocyte biology, it has become clear that normal glomerular filtration relies heavily on properly functioning podocytes. Although many proteins are involved in podocyte function, NEPHRIN and NEPH1 are key proteins that constitute the building blocks of the slit diaphragm and are critical for podocyte stability and integrity (1, 2). Of importance, mutations or genetic deletions of NEPHRIN and NEPH1 lead to dysfunctional podocytes, which, in turn, result in loss of renal filtration function (2, 3, 4, 5). Although several studies suggest that the extracellular domains of NEPHRIN and NEPH1 have a structure-based function where their spatial arrangement provides integrity to the slit diaphragm, their intracellular domains were shown to initiate signaling cascades leading to actin cytoskeletal changes in podocytes (1, 6, 7). This suggests that NEPHRIN and NEPH1 undergo activation that is driven by phosphorylation prior to initiation of downstream signaling. However, the mechanism(s) behind phosphorylation of these proteins remain unknown. Moreover, without the knowledge of a specific ligand that induces phosphorylation of these proteins, the primary function assigned to their extracellular domains remains structural organization of the slit diaphragm. In this study, a receptor-based phosphorylation mechanism was identified in which engagement of the extracellular domains of NEPHRIN and NEPH1 with the ligand hepatocyte growth factor (HGF) induced their phosphorylation. We identified SH2 domain–containing protein tyrosine phosphatase-2 (SHP-2) as a novel phosphatase for these proteins. Furthermore, functional studies using *in vitro* and *ex vivo* models of injury demonstrate that, in response to injury, recovery is initiated in an HGF-dependent manner, which involves ligand-based phosphorylation of NEPHRIN and NEPH1 leading to actin cytoskeletal reorganization and podocyte repair.

## Results

### SHP-2 is a novel binding partner for NEPH1

**Figure 1:**
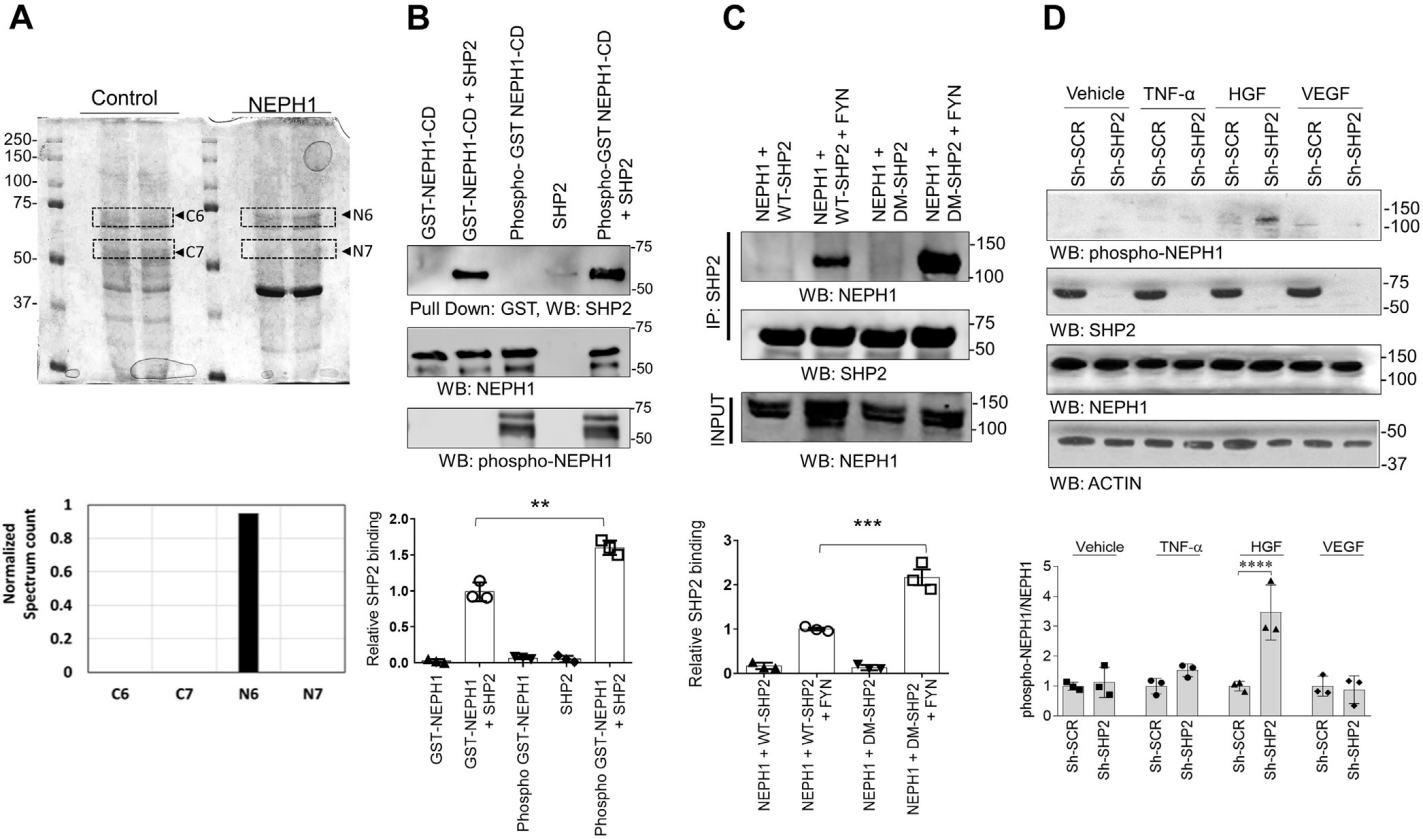
**The phosphatase SHP-2 binds NEPH1.***A*, immunoprecipitation of endogenous NEPH1 from podocytes followed by mass spectrometry analysis identified SHP-2 (N6) as a novel NEPH1 interacting protein**.** The plot shows the normalized spectrum values of four different bands excised between 75 and 50 kDa (highlighted as C6 and C7 from IgG controls and N6 and N7 from NEPH1 antibody) derived by using Scaffold software (Proteome Software, Inc). *B*, direct binding was evaluated by mixing purified phosphorylated GST-NEPH1-Cytoplasmic Domain (NEPH1-CD, produced in TKB1 cells) with purified recombinant SHP-2, which showed increased binding of SHP-2 with phosphorylated GST-NEPH1-CD. *C*, NEPH1 was coexpressed with SHP-2 or the substrate trapping SHP-2 mutant (DM SHP-2) with or without the phosphorylating agent FYN. To evaluate NEPH1 binding, SHP-2 was immunoprecipitated from the cell lysate and Western blotted (WB) with NEPH1 antibody. This demonstrated that NEPH1 bound SHP-2 in the presence of FYN. Significantly enhanced binding was observed with DM SHP-2 indicating that NEPH1 is a SHP-2 substrate. *D*, control podocytes and podocytes with stable SHP-2 knockdown were treated with growth factors (TNF-α, HGF, and VEGF), and the phosphorylation of endogenous NEPH1 was evaluated using phospho-NEPH1 antibody. NEPH1 phosphorylation was significantly increased only following HGF stimulation of SHP-2 KD podocytes. Experiments *B*–*D* were performed in triplicate, repeated three times with similar results, and representative images of the results are presented in the figure. Data are presented as mean ± SEM, and *p*-values were calculated using the Sidak's multiple comparisons test (two-way ANOVA). ∗∗*p* ≤ 0.01, ∗∗∗*p* ≤ 0.001, ∗∗∗∗*p* ≤ 0.0001. SCR, scrambled.

To identify novel NEPH1-binding proteins we performed coimmunoprecipitation experiments. The proteins that immunoprecipitated with NEPH1 were analyzed by mass spectrometry. Analysis of the Neph1-binding proteins was performed using the Scaffold proteomics software, and 123 proteins were identified. SHP-2, a product of the tyrosine-protein phosphatase non-receptor type 11 (PTPN11) gene, was one of these proteins and had previously been linked to NEPH1 (8) (Fig. 1*A*). By mixing recombinant purified proteins we demonstrated a direct interaction between the cytoplasmic domain of NEPH1 and SHP-2 (Fig. 1*B*). Since SHP-2 binds NEPHRIN in a phosphorylation-dependent manner (8), we investigated whether NEPH1 phosphorylation also enhanced SHP-2 binding. Similar to NEPHRIN, phosphorylation of NEPH1 can be accomplished either by the treatment of cells (stable HEK293 NEPH1-overexpressing cells) with pervanadate (6, 9) or by coexpressing with FYN kinase. In Figure 1*C* we show that FYN kinase significantly increases NEPH1 and SHP-2 binding. To further determine if NEPH1 is a substrate for SHP-2, we tested the binding of NEPH1 with a substrate trapping SHP-2^DM^ mutant (10, 11). SHP-2 is a phosphatase (12), and the substrate trapping SHP-2^DM^ mutant displayed a much higher ability to bind phosphorylated NEPH1 than the wildtype SHP-2 (Fig. 1*C*), indicating a functional interaction between the two proteins.

### HGF, but not other growth factors, induces NEPH1 phosphorylation

Under physiologic conditions, detection of the phosphorylated form of a protein (typically only 5%–10% of the total protein) can be challenging owing to the presence of phosphatases. Since SHP-2 appeared to be a potent phosphatase for NEPH1, we hypothesized that the phosphorylation (ligand-induced) of endogenous NEPH1 would be suppressed in the presence of SHP-2. We therefore generated stable SHP-2 knockdown (KD) human podocytes that are known to endogenously express NEPH1 and tested the level of NEPH1 phosphorylation following exposure to various growth factors using a NEPH1-specific phosphoantibody (6, 13, 14). Phosphorylation of endogenous NEPH1 was only visible in SHP-2 knockdown podocytes treated with HGF (Fig. 1*D*).

### SHP-2 dephosphorylates NEPHRIN and NEPH1

**Figure 2:**
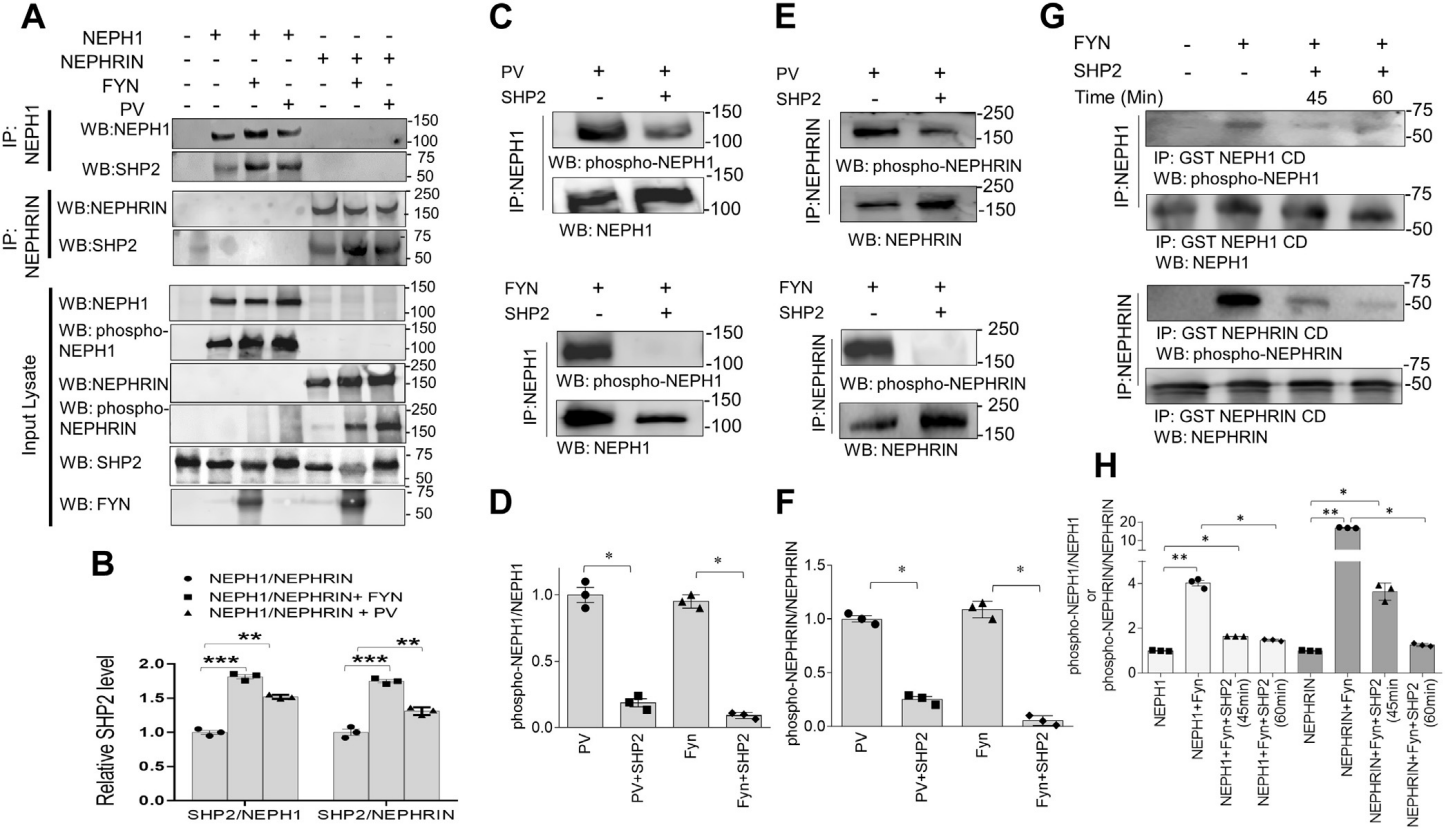
**SHP-2 binds NEPH1 and NEPHRIN and acts as a phosphatase.***A* and *B*, NEPH1 or NEPHRIN were phosphorylated by either coexpressing with FYN or treating with pervanadate and were immunoprecipitated with their respective antibodies. Western blotting of the immunoprecipitated complexes showed increased SHP-2 binding to phosphorylated NEPHRIN and NEPH1. Data are presented as mean ± SEM, and *p*-values were calculated using the Kruskal–Wallis one-way analysis of variance. *C*–*F*, HEK293 cells expressing NEPHRIN or NEPH1 were treated with PV followed by immunoprecipitation of NEPH1 and NEPHRIN (set 1). Simultaneously, NEPH1 and NEPHRIN were overexpressed in HEK293 cells and the proteins were immunoprecipitated using their respective antibodies and incubated with purified FYN (500 ng) (set 2). Recombinant SHP-2 protein was then added to the immunoprecipitated complexes (both sets) and the extent of dephosphorylation was measured by their respective phosphoantibodies. Data are presented as mean ± SEM, and *p*-values were calculated using the Mann–Whitney (nonparametric) test, one-tailed. *G* and *H*, purified recombinant GST-NEPH1 and GST-NEPHRIN cytoplasmic domain (CD) proteins were phosphorylated by incubating with recombinant active FYN immobilized on nickel beads. Post phosphorylation, FYN beads were removed and the phosphorylated GST-NEPH1 cytoplasmic domain (GST-NEPH1 CD) and GST-NEPHRIN CD proteins were incubated with purified recombinant SHP-2 for the indicated times and Western blot was performed with the respective phosphoantibodies, which confirmed SHP-2-mediated dephosphorylation. Data are presented as mean ± SEM, and *p*-values were calculated using the Kruskal–Wallis one-way analysis of variance. ∗*p* ≤ 0.05, ∗∗*p* ≤ 0.005, ∗∗∗*p* ≤ 0.0005. All experiments were performed in triplicate and repeated three times with similar results, and representative images of the results are presented in the figure.

As shown in Figure 1*C*, FYN kinase increases binding of SHP-2 to NEPH1. We next confirmed that FYN and pervanadate increased the binding of SHP-2 to both NEPH1 and NEPHRIN. (Fig. 2, *A* and *B*). Although a previous report suggested that coexpression of SHP-2 with NEPHRIN enhanced its phosphorylation owing to its effect on FYN activation, a direct effect of SHP-2 on NEPHRIN was not evaluated (8). Since SHP-2 is a phosphatase, we hypothesized that SHP-2 directly dephosphorylates NEPH1 and NEPHRIN. To test this, we coexpressed NEPH1 or NEPHRIN with FYN in HEK293 cells and we also treated the NEPH1- and NEPHRIN-expressing stable cultured podocytes (2) with pervanadate and then immunoprecipitated the proteins with their respective antibodies. The immunoprecipitated complexes containing the phosphorylated NEPHRIN or NEPH1 were incubated with purified active recombinant SHP-2 in a phosphatase buffer. Significant dephosphorylation of both NEPHRIN and NEPH1 were noted in the presence of SHP-2 (Fig. 2, *C*–*F*). Subsequently, we incubated purified glutathione S-transferase (GST)-NEPH1 or GST-NEPHRIN cytoplasmic domains with purified active HIS-FYN (0.5 μg) that was immobilized on Ni-NTA beads. The resulting phosphorylated NEPHRIN and NEPH1 proteins were removed by centrifugation and incubated with purified active recombinant SHP-2 phosphatase, which resulted in the dephosphorylation of these proteins in a time-dependent fashion (Fig. 2, *G* and *H*). Collectively, these results demonstrate that SHP-2 is a phosphatase for NEPHRIN and NEPH1.

### HGF is a novel inducer of NEPH1 and NEPHRIN phosphorylation

**Figure 3:**
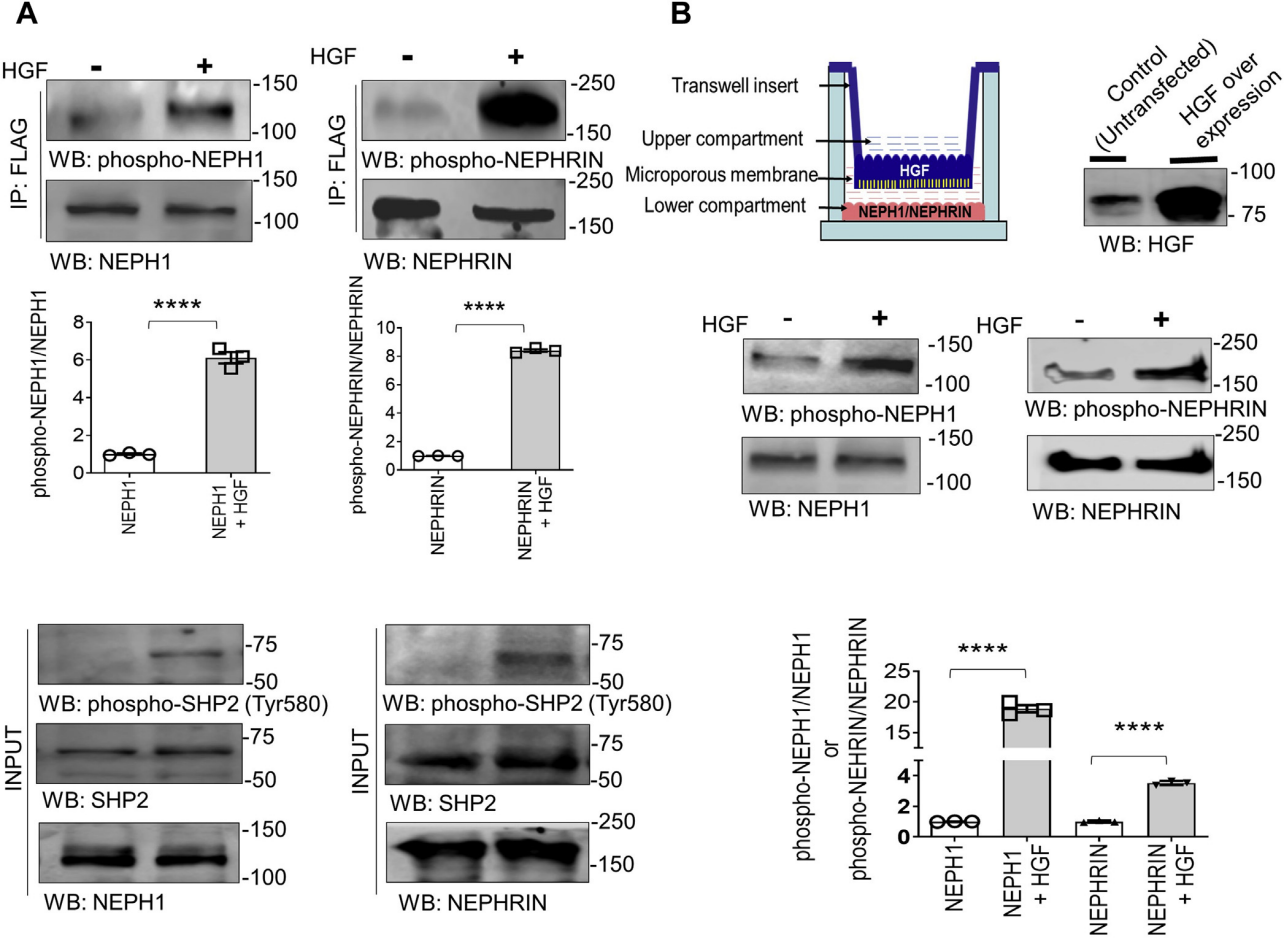
**HGF is a novel inducer of NEPHRIN and NEPH1 phosphorylation.***A*, NEPHRIN-FLAG and NEPH1-FLAG were coexpressed with HGF in HEK293 cells, and phosphorylation was analyzed using their respective phosphoantibodies. SHP2 was also phosphorylated in the presence of HGF. Data are presented as mean ± SEM, and *p*-values were calculated using a two-tailed Student’s *t* test. *B*, HGF-expressing HEK293 cells were grown on the Transwell filter and stable NEPH1- and NEPHRIN-expressing HEK293 cells were cultured on the plastic at the bottom of the well. NEPHRIN and NEPH1 phosphorylation was measured by lysing the cells in the well, and Western blot analysis was performed with their respective phosphoantibodies. Data are presented as mean ± SEM, and *p*-values were calculated using a two-tailed Student’s *t* test. ∗∗∗∗*p* ≤ 0.001. All experiments were performed in triplicate and repeated three times with similar results, and representative images of the results are presented in the figure.

HGF is an established activator of the mesenchymal epithelial transition (MET) receptor and SHP-2 (15, 16). Since the concept of NEPH1 and NEPHRIN phosphorylation by HGF is novel and may have significant biological and clinical implications, we investigated this further using two independent techniques. First, NEPHRIN and NEPH1 were coexpressed with HGF in HEK293 cells and the cell lysates were probed using Western blot with NEPHRIN- and NEPH1-specific phosphoantibodies, which showed that NEPHRIN and NEPH1 were phosphorylated in the presence of HGF (Fig. 3*A*). In addition, we performed a Transwell assay where HGF-overexpressing HEK293 cells were cultured on the Transwell filter and NEPHRIN- or NEPH1-overexpressing HEK293 cells were cultured on the bottom of the wells (Fig. 3*B*). Significant phosphorylation of NEPHRIN and NEPH1 was noted only when the Transwell filters contained HGF-expressing cells (Fig. 3*B*). These results demonstrate that NEPHRIN and NEPH1 phosphorylation can be induced in the presence of HGF.

### HGF can induce phosphorylation of NEPHRIN and NEPH1 in the absence of MET

**Figure 4:**
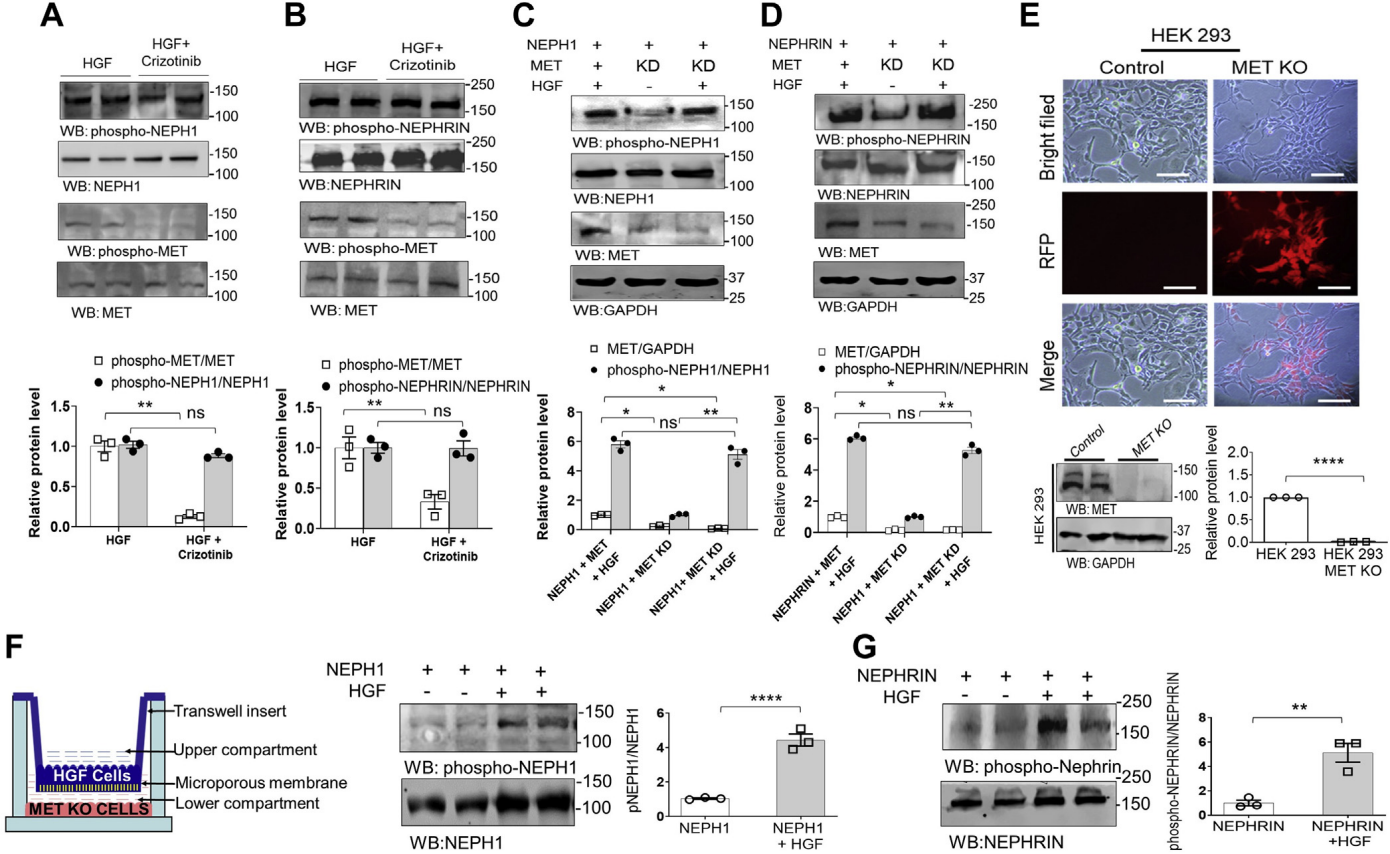
**The MET receptor is not required for HGF-induced NEPHRIN and NEPH1 phosphorylation.** Recombinant HGF (20 ng/ml) was added to HEK293 cells overexpressing (*A*) NEPH1-FLAG or (*B*) NEPHRIN-FLAG in the presence or absence of 100 nM Crizotinib, a MET inhibitor. Phosphorylation of NEPH1 and NEPHRIN was unchanged by the presence of Crizotinib. Data are presented as mean ± SEM, and *p*-values were calculated using the Kruskal–Wallis one-way analysis of variance. *C*, *D*, NEPH1-FLAG or NEPHRIN-FLAG were overexpressed in HEK293 cells with stable shRNA-mediated knockdown (KD) of the MET receptor. Recombinant HGF (20 ng/ml) was added to these cells, and NEPHRIN and NEPH1 phosphorylation in the cell lysate showed no change in phosphorylation following MET knockdown (KD). Data are presented as mean ± SEM, and *p*-values were calculated using the Kruskal–Wallis one-way analysis of variance. *E*, using CRISPR-Cas9, stable MET knockout HEK293 cells were generated and transfected with NEPHRIN- and NEPH1-expressing plasmids. Red fluorescent protein (RFP) is a marker for transfection and confirms the stable knockout of MET post puromycin selection. Data are presented as mean ± SEM, and *p*-values were calculated using a two-tailed Student’s *t* test. The scale bar represents 25 μm. *F* and *G*, using HGF-overexpressing cells grown on the Transwell filter and HEK293 MET knockout cells expressing NEPH1 or NEPHRIN growing on the plastic at the bottom of well (first panel, schematic), we demonstrate that NEPH1 and NEPHRIN phosphorylation occurs following complete loss of the MET receptor. Data are presented as mean ± SEM, and *p*-values were calculated using a two-tailed Student’s *t* test. ns, nonsignificant, ∗*p* ≤ 0.05, ∗∗*p* ≤ 0.01, ∗∗∗∗*p* ≤ 0.0001. All experiments were performed in triplicate and repeated three times with similar results, and representative images of the results are presented in the figure.

Since HGF is known to be a potent activator of the MET receptor (17, 18), we investigated if MET was directly or indirectly involved in NEPHRIN and NEPH1 phosphorylation. We first used a MET receptor inhibitor (Crizotinib) (19). Crizotinib when added to NEPHRIN- and NEPH1-overexpressing HEK-293 cells was unable to attenuate the HGF-induced phosphorylation of NEPHRIN and NEPH1 (Fig. 4, *A* and *B*), indicating that MET was not required for NEPHRIN and NEPH1 phosphorylation. To further rule out MET involvement in NEPHRIN and NEPH1 phosphorylation, we performed two additional experiments. First, we generated HEK293 cells overexpressing NEPHRIN or NEPH1 and containing a stable knockdown of the MET receptor, followed by the addition of HGF to induce phosphorylation. As shown, the MET-KD cells displayed no change in HGF-mediated phosphorylation of NEPHRIN or NEPH1 (Fig. 4, *C* and *D*), suggesting that HGF induces NEPHRIN and NEPH1 phosphorylation independent of the MET receptor. Finally, we used the CRISPR-Cas system to generate stable MET knockout HEK293 cells (Fig. 4*E*) followed by overexpression of NEPHRIN and NEPH1 in these cells. Using the Transwell assay we demonstrated that HGF could still induce phosphorylation of NEPHRIN or NEPH1 even in the absence of the MET receptor (Fig. 4, *F* and *G*).

### HGF is a novel ligand that binds NEPHRIN and NEPH1 extracellular domains

**Figure 5:**
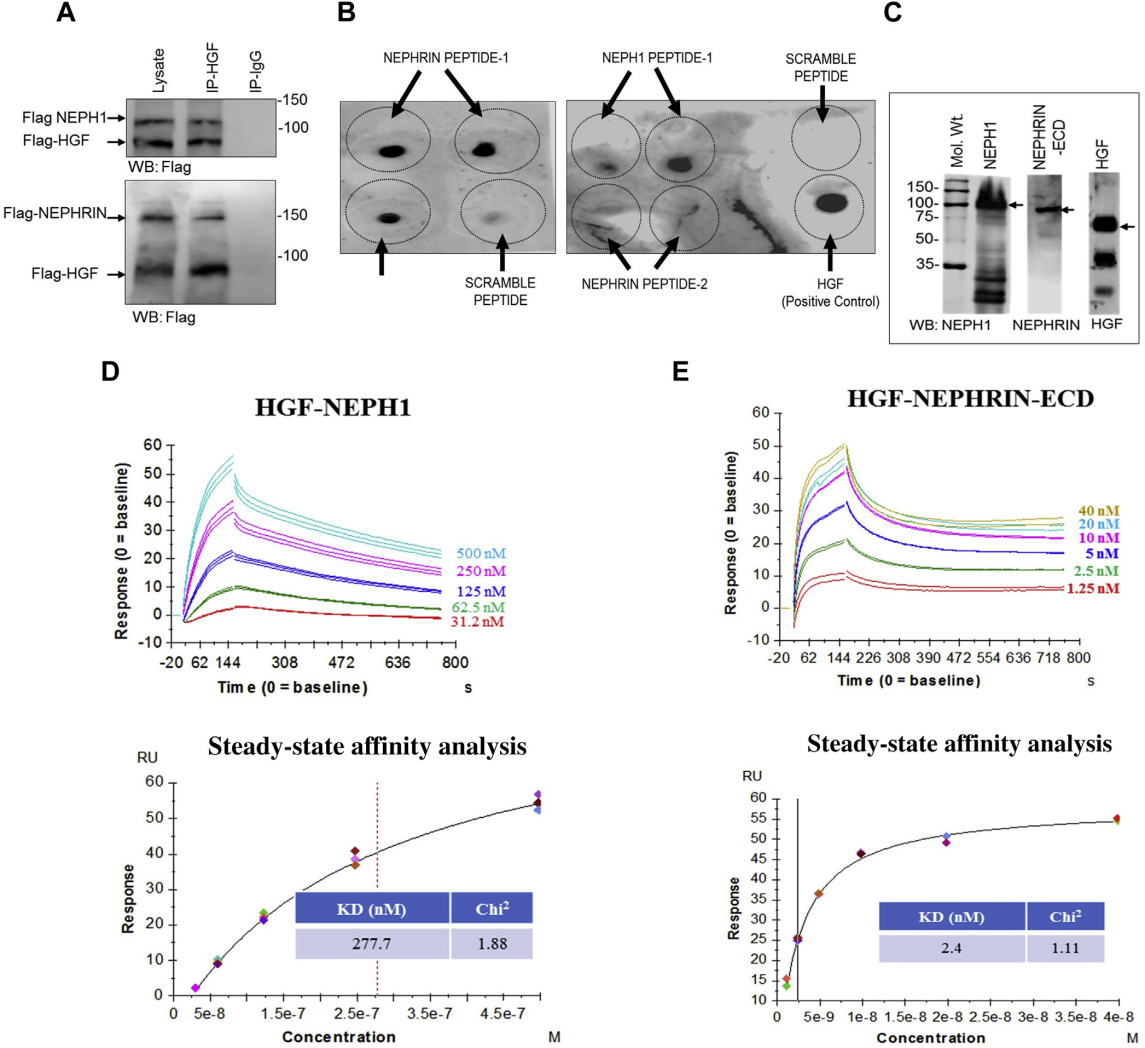
**HGF interacts with NEPHRIN and NEPH1.*A*, NEPHRIN-FLAG and NEPH1-FLAG were coexpressed with HGF-FLAG in HEK293 cells, respectively.** HGF was immunoprecipitated using the HGF antibody, and the immunoprecipitated complexes were analyzed for NEPHRIN-FLAG and NEPH1-FLAG binding by Western blot using the FLAG antibody. *B*, dot blot assay: Synthetic peptides corresponding to the HGF-binding regions of NEPHRIN and NEPH1 were spotted onto nitrocellulose membranes and probed with recombinant HGF. HGF binding was seen with the peptides NEPH1 Peptide-1 and NEPHRIN Peptide-1 but not with NEPHRIN Peptide-2 or control (scrambled) peptides. *C*–*E*, baculovirus-expressed NEPHRIN- extracellular domain (ECD) and NEPH1 (*C*) were mixed with HGF in the indicated amounts and subjected to surface plasmon resonance. Surface plasmon resonance analysis showed the concentration-dependent binding of NEPH1 (*D*) and NEPHRIN (*E*) with HGF. The steady-state affinity analysis and the calculated respective KD values have been shown for NEPH1 (*D*, *lower panel*) and NEPHRIN (*E*, *lower panel*).

**Figure 6:**
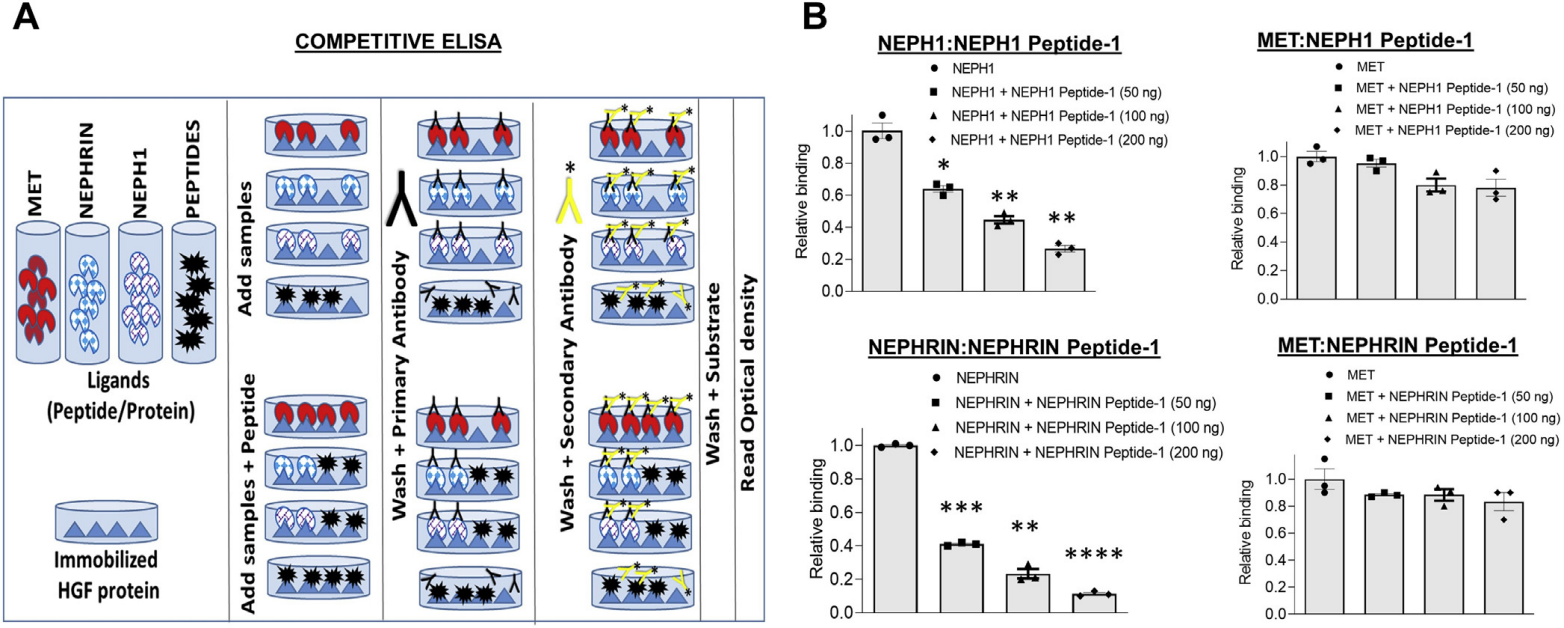
**The HGF-binding site for MET does not overlap with the HGF-binding sites for NEPH1 and NEPHRIN.***A*, schematic for the competitive ELISA. HGF was immobilized on the wells of an ELISA plate, and its binding to NEPH1, NEPHRIN, and MET alone and in combination with NEPH1 peptide-1 and NEPHRIN peptide-1 was analyzed. *B*, quantification of the data. All comparisons are with NEPH1 or NEPHRIN alone. Data are presented as mean ± SEM, and *p*-values were calculated using a two-tailed Student’s *t* test. ∗*p* ≤ 0.05, ∗∗*p* ≤ 0.01, ∗∗∗*p* ≤ 0.001, ∗∗∗∗*p* < 0.0001.

If HGF acts as a ligand then it should interact extracellularly with NEPHRIN and NEPH1. To test this, we first coexpressed HGF with NEPHRIN or NEPH1 and evaluated their binding through coimmunoprecipitation. This showed that both NEPHRIN and NEPH1 interacted with HGF (Fig. 5*A*). To determine whether the interaction is direct, we performed two independent experiments. First, using sequence homology and a molecular modeling approach, with the HGF-binding site of the MET receptor as a template, we identified potential binding regions in the extracellular domains of NEPHRIN and NEPH1. These analyses revealed IgG3 in NEPH1 and IgG2 in NEPHRIN as putative HGF interacting domains (Fig. S1). These regions were highly conserved among multiple species (Fig. S2). The peptides from these regions were synthesized and tested in a dot blot assay, where peptides were immobilized and probed with recombinant HGF. The results demonstrated that HGF interacted with NEPH1 Peptide-1 and NEPHRIN Peptide-1 but not with NEPHRIN Peptide-2 (Fig. 5*B*). To further validate these results recombinant mammalian NEPHRIN and NEPH1 proteins were generated. As shown in Figure 5*C*, we used an SF9 insect cell line and the Baculoviral Expression system to express mammalian His-FLAG-NEPH1- full length (FL) and His-FLAG-NEPHRIN- extracellular domain (ECD). The purified recombinant NEPHRIN and NEPH1 proteins were mixed with commercially obtained purified HGF (Fig. 5*C*), and surface plasma resonance was employed to evaluate their binding. The results showed a concentration-dependent direct interaction of HGF with NEPHRIN and NEPH1 (Fig. 5, *D* and *E*). Of interest, the interaction of HGF with NEPH1 was in the mid nanomolar range (K_D_ = 278 nM) (steady-state analysis, Fig. 5*D*, lower panel), whereas the interaction with NEPHRIN was in the low nanomolar range (K_D_ = 2.4 nM) (steady-state analysis, Fig. 5*E*, lower panel), indicating that HGF binds NEPHRIN with a much higher affinity (Fig. 5, *D* and *E*). To further test if the binding sites of HGF for NEPHRIN and NEPH1 overlap with MET we performed a competitive ELISA. HGF was immobilized on individual wells in an ELISA plate and HGF binding to MET, NEPH1, or NEPHRIN was evaluated in the presence of increasing concentrations of HGF-binding NEPH1 and NEPHRIN peptides (Fig. 6, *A* and *B*). Although NEPH1 Peptide-1 inhibited the binding of NEPH1 to HGF and NEPHRIN Peptide-1 inhibited the binding of NEPHRIN to HGF, these peptides failed to inhibit the binding of HGF to MET. These data confirm that the HGF-binding sites for NEPH1 and NEPHRIN do not overlap with the HGF-binding site for MET.

### HGF induces podocyte recovery from injury in *in vitro* and *ex vivo* models

**Figure 7:**
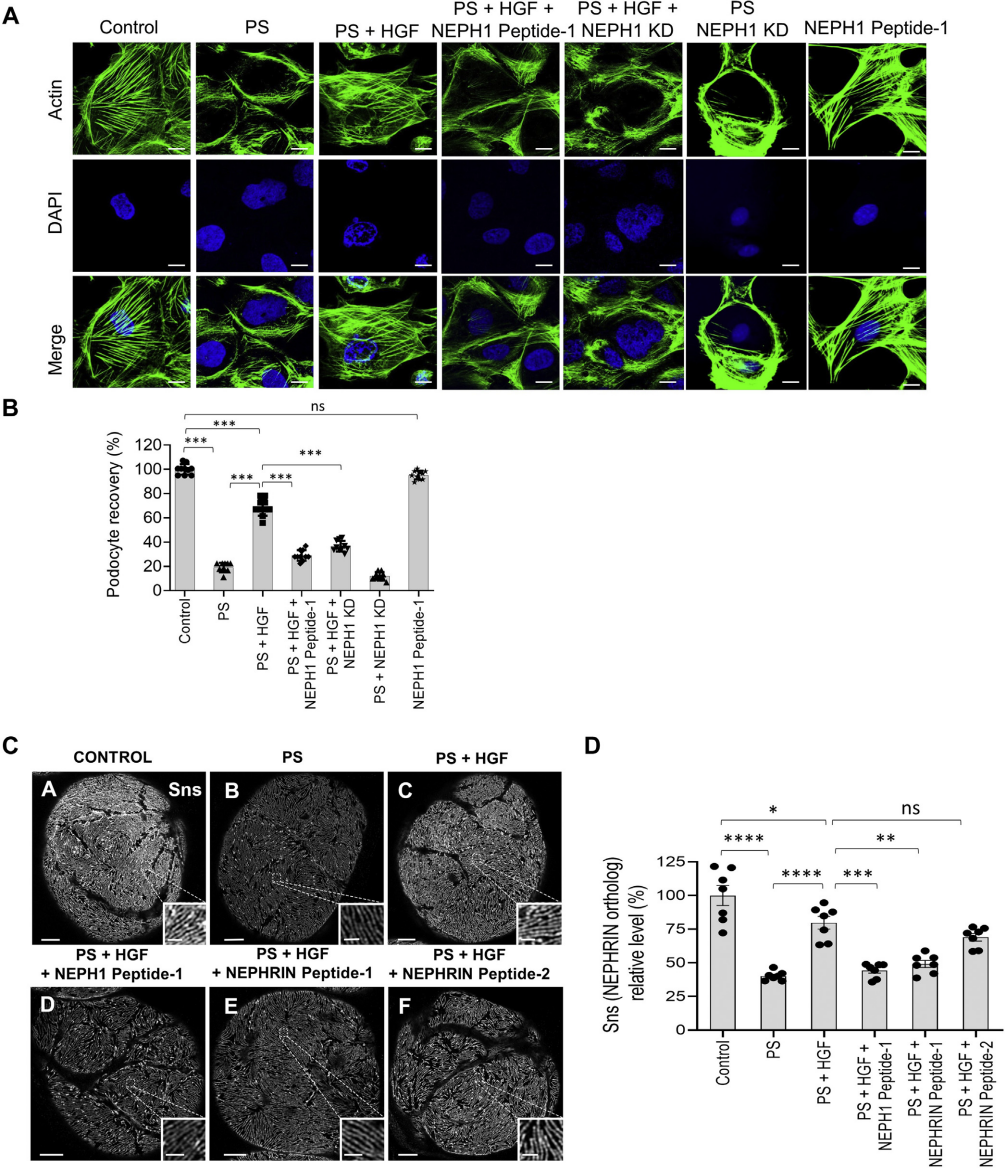
**HGF treatment repairs podocytes/nephrocytes in a NEPH1- and NEPHRIN-dependent fashion.***A* and *B*, cultured human podocytes were treated with protamine sulfate (PS), and actin cytoskeleton (*green*) disorganization was visualized by phalloidin staining. To induce recovery, HGF (50 ng/ml) was added to the PS-treated podocytes. The addition of NEPH1 inhibitory peptide blocked HGF-induced recovery. KD of NEPH1 also prevented HGF-induced recovery. Ten cells per experimental condition were evaluated from three experimental replicates. The scale bar represents 25 μm. Data are presented as mean ± SEM, and *p*-values were calculated using Tukey's multiple comparisons test (one-way ANOVA). *C* and *D*, Sns (*Drosophila* ortholog of NEPHRIN) staining of *Drosophila* nephrocytes treated with PS in the presence or absence of HGF and NEPHRIN or NEPH1 peptides. Decreased Sns staining was noted in PS-treated nephrocytes, which was rescued by treatment with HGF. Addition of HGF-interacting NEPH1 Peptide-1 and NEPHRIN Peptide-1, but not NEPHRIN Peptide-2, blocked the rescue by HGF. The scale bar represents 5 μm; scale bar for *insets* represents 1 μm. For quantification, seven nephrocytes from three flies for each condition were analyzed. Data are presented as mean ± SEM, and *p*-values were calculated using the Tukey's multiple comparisons test (one-way ANOVA). ns, nonsignificant, ∗*p* ≤ 0.05, ∗∗*p* ≤ 0.01, ∗∗∗*p* ≤ 0.001, ∗∗∗∗*p* ≤ 0.0001.

Increased plasma levels of HGF have been reported in various diseases (17, 18, 20, 21). Since HGF functions as an injury-induced effector for tissue repair, we hypothesized that HGF is involved in podocyte repair following injury. To test this, cultured human podocytes overexpressing NEPH1 were injured with protamine sulfate (PS), which results in severe actin cytoskeletal disorganization (22). Addition of recombinant HGF resulted in significant recovery of the podocyte actin cytoskeleton (Fig. 7, *A* and *B*). Since the cultured podocytes express measurable amounts of endogenous NEPH1, but not NEPHRIN, addition of the NEPH1 peptide (that binds HGF), or use of NEPH1 knockdown podocytes (Fig. S3, *A* and *B*), blocked the HGF-induced recovery (Fig. 7, *A* and *B*). In parallel, we performed a similar experiment with NEPHRIN-overexpressing podocytes, which also showed HGF-induced recovery of podocytes from injury that was similarly blocked by NEPHRIN Peptide-1 (Fig. S4, *A* and *B*).

**Figure 8:**
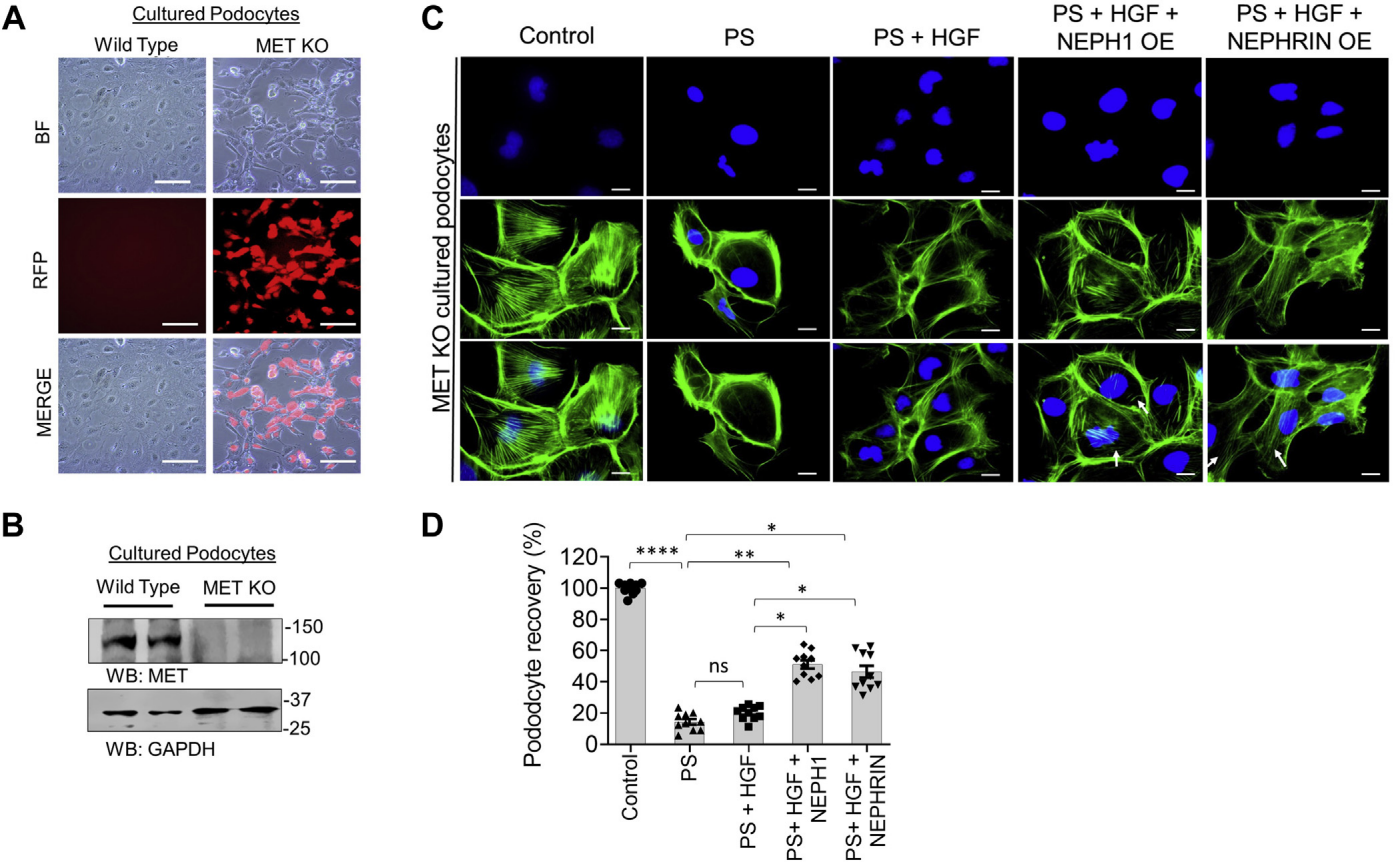
**NEPH1/NEPHRIN augments HGF-mediated recovery in protamine sulfate (PS)-induced injury in MET knockout podocytes.***A* and *B*, using CRIPR-Cas9 MET knockout podocytes were generated. Red fluorescent protein (RFP) is a marker for transfection and confirms the stable knock out of MET post puromycin selection. Western blot for the MET knockout and control podocyte cell lysates. *C* and *D*, MET knockout podocytes were transfected with NEPHRIN- and NEPH1-expressing plasmids and then treated with PS and the actin cytoskeletal (*green*) disorganization was visualized by phalloidin staining. To induce recovery, HGF (50 ng/ml) was added to the PS-treated podocytes. Podocytes transfected with NEPHRIN and NEPH1 showed significantly improved recovery with HGF. Ten cells per experimental condition were evaluated from three experimental trials. The scale bar represents 25 μm. Data are presented as mean ± SEM, and *p*-values were calculated using the Kruskal–Wallis test one-way analysis of variance. ns, nonsignificant, ∗*p* ≤ 0.05, ∗∗ *p* ≤ 0.01, ∗∗∗*p* ≤ 0.001.

To further study HGF-induced recovery in an *ex vivo* model system, *Drosophila* nephrocytes were used. Nephrocytes isolated from *Drosophila* were subjected to PS treatment and stained with antibody to sticks-and-stones (SNS, the *Drosophila* ortholog of NEPHRIN) to evaluate the extent of injury. SNS is a member of the immunoglobulin superfamily that is essential for myoblast fusion and formation of a slit diaphragm-like structure in insect nephrocytes (23). We observed a significant decrease in SNS protein in nephrocytes in response to injury, which was rescued by HGF treatment (Fig. 7, *C* and *D*). Furthermore, the HGF-interacting peptides, NEPH1 Peptide-1 and NEPHRIN Peptide-1, blocked the rescue effect of HGF indicating that recovery is mediated in a NEPH1- and NEPHRIN-dependent manner. In contrast, NEPHRIN Peptide-2, which does not interact with HGF, did not block the recovery following HGF. Collectively, these results demonstrate that HGF-induced NEPH1 and NEPHRIN phosphorylation participate in the recovery of podocytes following injury. Since a previous report suggested that the MET receptor is involved in podocyte recovery (24), we wanted to know if HGF-induced NEPHRIN and NEPH1 phosphorylation could serve as an alternate mechanism for podocyte recovery from injury. Therefore, CRISPR-Cas9-mediated MET knockout cultured podocytes (Fig. 8, *A* and *B*) were transfected with lentiviral particles expressing either NEPH1 or NEPHRIN and injured with PS followed by HGF-induced recovery. Although recovery was minimal in MET knockout podocytes, significant recovery following HGF addition was observed in MET knockout podocytes where either NEPH1 or NEPHRIN was overexpressed (Fig. 8, *C* and *D*). Thus, our *in vitro* and *ex vivo* results indicate that NEPHRIN, NEPH1, HGF, and SHP-2 participate in podocyte repair following injury.

## Discussion

Determining factors that regulate podocyte repair are critically important for the identification of much needed therapies to treat podocytopathies. NEPHRIN and NEPH1 are slit diaphragm proteins that are critical for podocyte function, and genetic defects in these proteins lead to renal dysfunction in mice and humans (7, 14, 25). The prevailing dogma indicates that the extracellular domains of NEPHRIN and NEPH1 constitute the structural framework of the slit diaphragm, thereby maintaining its structural integrity (25, 26, 27). It is known that NEPHRIN and NEPH1 transduce outside-in signaling (6, 9, 28); however, the mechanisms for such transduction remain unknown. In this study we present evidence that NEPHRIN and NEPH1 have receptor-like properties, where they can be phosphorylated (activated) in a ligand-based fashion by HGF and dephosphorylated (deactivated) by the phosphatase SHP-2. Previous biochemical and genetic studies showed that Src family kinases mediated tyrosine phosphorylation of the NEPHRIN and NEPH1 cytoplasmic domains in podocytes to induce subsequent downstream signaling events that led to actin cytoskeletal reorganization (8, 29). However, the pathways that regulate the phosphorylation of NEPHRIN and NEPH1 remained unknown (6, 9, 26). The data presented here also show that SHP-2 acts as a phosphatase that directly dephosphorylates NEPH1 and NEPHRIN. Since injury to podocytes is known to induce NEPHRIN and NEPH1 phosphorylation (3, 8, 30), we hypothesized that NEPHRIN and NEPH1 phosphorylation may require the downregulation of SHP-2 expression. Indeed, mRNA profiling of cultured podocytes injured by the puromycin amino nucleoside (Gene Expression Omnibus accession number GSE124622 (31)) showed a 5-fold reduction in SHP-2 expression (Fig. S5). Collectively, these results are consistent with a role for SHP-2 in regulating NEPHRIN and NEPH1 dephosphorylation. Furthermore, this may explain the protective effect observed in SHP-2 KO mice (32), where loss of SHP-2 could allow for persistent phosphorylation of NEPHRIN and NEPH1, leading to more efficient repair of the podocyte actin cytoskeleton.

Although our results highlight the dephosphorylation (deactivation) mechanism for NEPHRIN and NEPH1, the biochemical stimulus inducing phosphorylation of NEHPRIN and NEPH1 also had not been identified. We observed that HGF, which induces SHP-2 phosphorylation *via* the MET receptor, also induced phosphorylation of NEPHRIN and NEPH1. We used multiple biochemical approaches to establish HGF as the inducer of NEPHRIN and NEPH1 phosphorylation (Figs. 1*D* and 3). HGF acts as an injury-induced effector for tissue repair in both a paracrine and endocrine manner (33). Our Transwell assays and the specific antibodies we used demonstrated that HGF can induce NEPHRIN and NEPH1 phosphorylation at tyrosine 1176 or 1193 and tyrosine 637 or 638, respectively, in an endocrine fashion (Fig. 3*B*). Although the systemic deletion of HGF in mice induces early embryonic lethality (18, 34), podocyte-specific knockout of its receptor c-MET did not result in structural or physiological loss of renal function (35). Using multiple approaches including a MET inhibitor, MET KD with shRNA, and MET KO using CRISPR, it is clear that HGF-induced NEPHRIN and NEPH1 phosphorylation can occur in the absence of MET.

**Figure 9:**
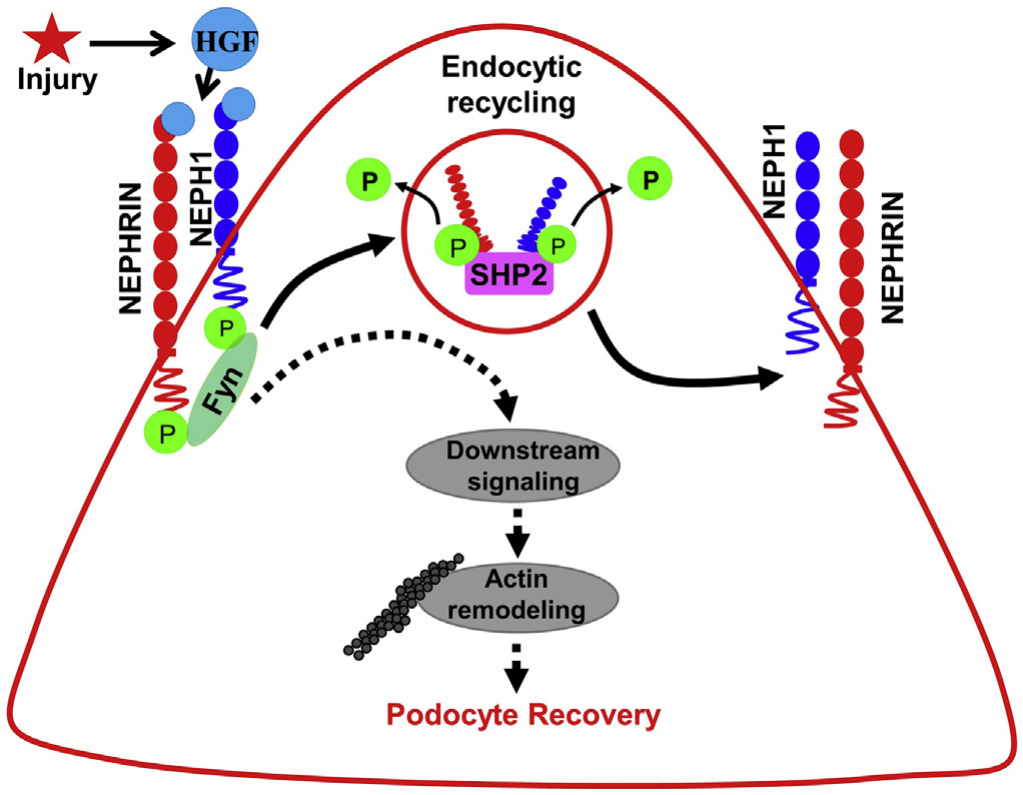
**Schematic of the phosphorylation (activation) and dephosphorylation (deactivation) mechanisms for NEPHRIN and NEPH1 in podocytes.** In response to injury, HGF initiates the recovery process by phosphorylating NEPHRIN and NEPH1, which induces actin cytoskeleton reorganization leading to podocyte repair. To maintain homeostasis, subsequent dephosphorylation is mediated in a SHP2-dependent manner.

To establish HGF as a NEPHRIN and NEPH1 ligand, we first demonstrated a direct interaction between HGF and NEPHRIN/NEPH1. We then showed that HGF interacted with NEPHRIN and NEPH1 at highly conserved specific sites in the extracellular IgG domain 2 of NEPH1 and on the IgG domain 3 of NEPHRIN. Of interest, the binding affinity of HGF to NEPHRIN was 20-fold higher than that of NEPH1, which is comparable with the reported affinity of HGF for the MET receptor (K_D_ = 0.2–0.3 nM (36)). Understanding which physiological functions are related to these different interactions/affinities will require further investigation (24, 37, 38, 39). Since HGF has renoprotective properties, we tested *in vitro* and *ex vivo* models of podocyte injury. PS treatment of cultured human podocytes and *Drosophila* nephrocytes induced actin cytoskeleton disorganization (40) and loss of SNS, the NEPHRIN ortholog (41), respectively. HGF induced the recovery of podocytes from injury, which occurred in a NEPHRIN- and NEPH1-dependent fashion. In response to injury, there is redistribution of proteins NEPHRIN and NEPH1 from the cell membrane to the cytoplasm (26) in a phosphorylation-dependent manner. Phosphorylation (activation) and dephosphorylation (deactivation) of NEPH1 and NEPHRIN is a dynamic process; however, the exact sequence of these events remains unclear. It has been established that NEPH1 and NEPHRIN phosphorylation is a proximal event that occurs both during development and following podocyte injury (42). A major obstacle in investigating the relevance of NEPH1 and NEPHRIN phosphorylation following injury has been the lack of understanding of the molecular mechanisms that regulate their phosphorylation. Although in this study we demonstrate HGF-induced NEPHRIN and NEPH1 phosphorylation, we also demonstrate the dephosphorylation of these proteins catalyzed by the phosphatase SHP-2. Although additional experimentation is required to understand the exact mechanism(s) and sequence whereby SHP-2 dephosphorylates NEPHRIN and NEPH1, our experiments suggest that SHP-2 participates in the recovery phase where dephosphorylation of NEPHRIN and NEPH1 is critical for translocating these proteins back to the cell membrane. Indeed, a decrease in NEPHRIN phosphorylation has been noted during the late recovery phase (27, 42). A clear understanding of the role of SHP-2 in NEPHRIN and NEPH1 activation/deactivation dynamics will require further investigation and will be the subject of our further studies. Overall, these data highlight a novel mechanism whereby renoprotective signals in podocytes are propagated *via* HGF-induced phosphorylation of NEPHRIN and NEPH1 (Fig. 9). In *Drosophila* nephrocytes we hypothesize that injury leads to an increase in HGF and phosphorylation of SNS, which results in redistribution of SNS from the membrane to the cytoplasm leading to the decreased signal observed in Figure 7. SHP2 then dephosphorylates SNS, and dephosphorylated SNS translocates back to the membrane.

Unlike the MET receptor tyrosine kinase (43), NEPHRIN and NEPH1 do not have kinase activity, and therefore, the complete mechanism through which HGF binding results in NEPHRIN and NEPH1 phosphorylation remains unclear. Although speculative, it is likely that HGF binding induces a conformational change in NEPHRIN and NEPH1, which results in the recruitment of the Src Family Kinase FYN to the intracellular region leading to phosphorylation. Another possibility is that NEPHRIN and NEPH1 act as coreceptors for a yet to be identified tyrosine kinase receptor that transphosphorylates these proteins. Detailed structural and exploratory analyses of these interactions may provide further mechanistic clues and will be the subject of future studies.

## Experimental procedures

### Plasmids, antibodies, and reagents

Full-length *Mus musculus* NEPHRIN and NEPH1 cDNAs were cloned using standard PCR cloning procedure as described (2). The HGF-expressing plasmid pCMV3-FLAG-HGF was procured from Sino Biological Inc. Substrate trapping SHP-2^DM^ (D425A/C459S) was provided by Dr Lazzara (44). Purified human recombinant HGF, VEGF, and TNFα were obtained from Sigma. Polyclonal purified antibodies to NEPH1, phospho-NEPH1, and NEPHRIN have been described (6, 45). GAPDH (Cat. No G8795) and anti-FLAG antibodies (Cat. No. F3165) and Puromycin were obtained from Sigma-Aldrich. Antibodies against SHP-2 (Cat. No. 3752S), MET (Cat No. 4560S), phospho-MET (Cat. No. 3077S), and FYN (Cat no. 4023) were obtained from Cell Signaling Technologies. NEPHRIN (Cat. No. ab227806) and Phospho-NEPHRIN (Cat No. ab80299, ab80298) were from Abcam. HGF antibody was obtained from Santa Cruz Biotechnology (Cat. No. Sc 13087). The SNS antibody was a gift from Dr Krahn (46). The cell transfection reagent Lipofectamine 2000 was purchased from Invitrogen (Cat. No. 11668019).

### Generation of lentiviral expression constructs for FLAG-NEPHRIN and FLAG-NEPH1

Standard PCR cloning techniques were used to generate mammalian expression plasmids encoding FLAG-tagged full-length NEPHRIN and NEPH1. PCR-amplified FLAG-NEPHRIN and NEPH1 were cloned at the EcoRI and EcoRI-Sal1 sites in the retroviral pBABE-puro vector, respectively, as described (2, 47). The FLAG tag was inserted between the signal peptide and the start of the extracellular domains of NEPHRIN and NEPH1. The fidelity of the constructs was determined by restriction digestion and DNA sequencing. Retroviruses overexpressing FLAG-NEPH1 and FLAG-NEPHRIN were generated by transfection of the respective plasmids into Phoenix cells according to the manufacturer’s instructions. Cultured podocytes and HEK293 cells were transfected with these viruses, and expression of FLAG-NEPHRIN and FLAG-NEPH1 was evaluated by Western blotting. Transfected cells were grown in 2.5 μg/ml of puromycin-containing medium for the selection of stably transfected cells. Details for the production of retroviruses and the transfection of podocytes to generate stable cell lines have been described (2).

### Cloning, expression, and purification of recombinant FLAG-NEPH1 full length and FLAG-NEPHRIN-extracellular domain

To study the *in vitro* biochemistry and kinetics of the interactions of NEPH1 and NEPHRIN with HGF, the baculovirus expression system was used. The Bac-to-Bac Baculoviral Expression system (Invitrogen, Catalog #10359-016) employing the SF9 insect cell line was used to express FLAG-NEPH1-FL and FLAG-NEPHRIN-ECD. The recombinant viral vector containing the gene insert of NEPH1 or NEPHRIN (rBacmid) was produced by transposition events in the *Escherichia coli* (DH10) host strain and blue-white selection was performed and white colonies picked. The insertion was confirmed by PCR and DNA sequencing. Following confirmation, the rBacmid was transfected into SF9 cells and the viral particles were used to infect SF9 cells for protein production. Cells were harvested to purify the HIS-FLAG-NEPH1-FL protein and HIS-FLAG-NEPHRIN ECD. The proteins were purified using a Ni-NTA affinity column. Column-bound proteins were eluted using 200 mM Imidazole on the AKTA FPLC system as described (47).

### Cell culture growth and treatments

The immortalized human podocyte cell line was obtained from Dr Saleem (48) and cultured in RPMI 1640–based medium supplemented with 10% fetal bovine serum (Invitrogen), 2 g/l of sodium bicarbonate (NaHCO_3_), insulin-transferrin-selenium supplement (Sigma-Aldrich), and 200 units/ml penicillin and streptomycin (Roche Applied Science) as described (6, 49). HEK293 cells were cultured in Dulbecco's modified Eagle's medium supplemented with 10% fetal bovine serum (Invitrogen) and 200 U/ml of penicillin and streptomycin (Invitrogen). Lipofectamine 2000 (Invitrogen) was used to perform transfection according to the manufacturer's protocol. HEK293 cells overexpressing FLAG NEPH1/NEPHRIN were plated and grown to 90% confluency and then serum starved for 2 h. Cells were then treated with fresh pervanadate (1 mM) for 45 min at 37 °C as described (50). Vehicle-treated cells were used as controls. After pervanadate treatment, cells were lysed in RIPA buffer supplemented with phosphatase and protease inhibitors. Protein estimation from each lysate was performed using the BCA protein kit, and equivalent amounts of lysates were subjected to SDS-PAGE and immunoblot analysis.

### *In vitro* phosphatase assays

To evaluate dephosphorylation of NEPHRIN and NEPH1 by SHP-2, GST-NEPH1-CD (cytoplasmic domain) or GST-NEPHRIN-CD (2 μg each) was mixed with purified active HIS-FYN (0.5 μg) immobilized on Ni-NTA beads in the presence of 1 mM ATP in 1× Kinase Buffer (25 mM Hepes at pH 7.4, 5 mM MgCl_2_, 5 mM MnCl_2_, 100 mM NaCl, 1 mM ATP) for 30 min at 30 °C. The beads were removed by centrifugation, and the phosphorylated GST-NEPHRIN and GST-NEPH1 were mixed with recombinant purified active SHP-2 (Recombinant Human SHP-2; Cat. No.: 1894-SH; R&D Systems) in the presence of phosphatase buffer (10 mM Hepes, pH7.4, 0.1 mM EDTA, and 1 mM DTT) and incubated at 37 °C for 45 to 60 min. Reactions were stopped by adding sample loading buffer (Thermo Scientific, Cat. No. 39000) and heating at 95 °C for 5 min. The tyrosine phosphorylation of substrate proteins was analyzed by immunoblotting using phospho-NEPH1/NEPHRIN antibodies.

### Coimmunoprecipitation

Coimmunoprecipitations were performed as described with minor modifications (45). HEK 293T cells stably expressing FLAG-NEPH1 or FLAG-NEPHRIN were transiently transfected with HGF expressing pCMV-HGF plasmid using the Lipofectamine 2000 transfection agent following the manufacturer’s instructions. After 48 h of incubation, cells were washed twice with PBS and lysed in RIPA buffer (phosphate-buffered saline [PBS] containing 0.1% sodium dodecyl sulfate [SDS], 1% Nonidet P-40, 0.5% sodium deoxycholate, and 100 mM potassium iodide) with EDTA-free proteinase inhibitor mixture (Roche Molecular Biochemicals). Lysates were cleared by centrifugation at 10,000 rpm for 10 min at 4 °C. After centrifugation, cell lysates containing equal amounts of total protein were incubated with anti-HGF antibody or mice/rabbit IgG for 2 h at 4 °C, followed by the addition of 5 μl (packed volume) of protein G–coupled Agarose beads (ROCHE) and continued overnight incubation at 4 °C. Beads were then collected by centrifugation at 3000 rpm for 5 min at 4 °C, extensively washed with PBST, and resuspended in SDS gel loading buffer. The proteins were separated on a 10% SDS-polyacrylamide gel, transferred to a PVDF membrane, and analyzed by immunoblotting with the corresponding antibodies. Control samples were incubated with a nonspecific rabbit antiserum followed by the addition of protein G beads.

### Molecular modeling for predicting HGF-binding regions in NEPH1 and NEPHRIN

The MET region involved in high-affinity binding to the HGF-α chain is located in the immunoglobulin-like domains IPT 3 and 4 of the MET receptor (36). To predict the HGF-binding site in NEPH1 and NEPHRIN, sequences corresponding to IPT 3 and 4 of MET receptor were separately aligned to the sequences of NEPH1 and NEPHRIN using the sequence alignment tool BLAST (pBlast). Sequences with the highest identify and lowest gap were predicted to have a high HGF binding probability. Two HGF-binding regions for NEPHRIN from the IgG domain 2, PDITILLSGQTISDISANVNEGSQQKL (NEPHRIN-P1) and FTVEATARVTPRSSDNRQLLVCEASS (NEPHRIN-P2), and one region from the NEPH1 IgG 3 domain, QEGERVVFTCQATANPEIL (NEPH-P1), were predicted. To further confirm the binding region, an online server for protein–protein interactions, Z dock, was used. The extracellular domains of NEPH1 and NEPHRIN and the HGF-α chain were modeled using the protein fold recognition-based modeling server PHYRE2 as described (51). PHYRE2 used the following PDB templates to generate the models: for HGF, the template used was 4dur.2.A, X-ray crystal structure of full-length type ii human plasminogen (93% coverage, 100% confidence); for NEPHRIN it was 3b43A, X-ray crystal structure of titin (89% coverage, 100% confidence); for NEPH1 it was 3b43A, X-ray crystal structure titin (85% coverage, 100% confidence); and for MET it was 5L5C, the X-ray crystal structure of Plexin A1 full extracellular region (81% coverage, 100% confidence). The modeled proteins were used in Z dock (http://zdock.umassmed.edu/). The predicted model for the MET-HGF (α-chain), NEPHRIN-HGF (α-chain), and the NEPH1-HGF (α-chain) were found to bind with equivalent energies in the predicted regions.

### Recombinant proteins and peptides

Proteins, including GST-NEPH1 cytoplasmic domain (CD) and GST-NEPHRIN CD were expressed and purified from *E. coli* BL21 cells (Stratagene). Purified phosphorylated GST-NEPH1 was expressed and purified from TKB1 cells (Stratagene). Details of the expression and purification protocol have been described (9, 52). The predicted NEPHRIN and NEPH1 peptide sequences (NEPHRIN-P1, P2, and NEPH1-P1) described above were synthesized chemically using standard 9-fluorenylmethoxy carbonyl (Fmoc) solid-phase chemistry, and 2-chloro trityl resin was used as the solid support (PTI peptide synthesizer). Post synthesis, the peptides were cleaved using a cocktail of trifluoroacetic acid and scavengers and were purified to homogeneity using high-performance liquid chromatography (HPLC) (C18 semi prep and analytical columns attached to a Waters system). Identities of the purified peptides were confirmed by MALDI-TOF MS (Voyager).

### Dot blot assay

The NEPH1-P1 and NEPHRIN-P1 and -P2 peptides were used in a dot blot assay as described (47). Briefly, the test and control peptides (300 ng) were spot blotted in duplicate onto nitrocellulose membrane and allowed to air dry. The scrambled peptide was used as a negative control and purified recombinant HGF was used as a positive control. The membrane was blocked for 1 h using fat-free milk and then incubated with HGF (1 μg recombinant purified HGF in 5% BSA in PBS) at 4 °C and then immunoblotted with anti-HGF antibody.

### Surface plasmon resonance

The real-time binding experiment was performed at the Biacore Molecular Interaction Shared Resource (BMISR) Center using the Biacore T200 instrument. pH scouting of ligand (HGF) was performed, and pH 5.0 was selected for the immobilization. HGF was diluted in 10 mM acetate buffer at pH 5.0 (1:10 dilution, 0.01 μg/μl diluted concentration) and immobilized to a level of ∼2000 RU. HBS-P (0.1 M Hepes, 1.5 M NaCl, 0.5% v/v Surfactant P20) buffer was used as the immobilization running buffer. HGF was immobilized on the CM5 chip cell, and the instrument was programmed to perform a series of binding assays with increasing concentrations of FLAG-NEPH1-FL and FLAG-NEPHRIN-ECD. The flow rate of all the solutions was maintained at 50 μl/min. Surface regeneration and complete dissociation between the two proteins were achieved by using 2 M NaCl. A 1:1 kinetic model fitting was used for the analysis of sensorgrams and calculating the kinetic constants (association constant [Ka], dissociation constant [Kd], and equilibrium dissociation constant [K_D_]).

### HGF influx assay using Transwell coculture plates

HEK293 cells overexpressing HGF were cultured on the filters of six-well Transwell plates (0.4-μm pore; Corning), and on the plastic in the wells FLAG-NEPH1- or FLAG-NEPHRIN-overexpressing HEK293 cells were cultured. The control cells grown on the filters consisted of non-HGF-expressing HEK293 cells. The cells were harvested after 48 h and lysed, and their phosphorylation was assessed by Western blotting using phosphoantibodies for NEPH1 and NEPHRIN.

### Immunofluorescence microscopy

Podocytes stably expressing FLAG-NEPHRIN and FLAG-NEPH1 were plated and grown to 80% to 90% confluency on glass coverslips coated with collagen. The cells were then serum starved overnight, washed with PBS, and subjected to injury by incubating with 500 μg/ml of protamine sulfate (PS) for 8 h at 33 °C in serum-free media. Post PS treatment, PS was removed and cells were then incubated with and without recombinant human active HGF (cell culture grade, 50 ng/ml) in 2% serum containing RPMI media for 8 h at 33 °C. Control sets included no treatment, NEPH1 KD podocytes, or podocytes where coincubation with NEPH1 or NEPHRIN inhibitory peptides (HGF-binding peptides) was performed. The cells were washed with PBS and fixed with 4% paraformaldehyde (in 1× PBS), followed by permeabilization with 0.1% SDS. Immunostaining was performed using fluorescent-labeled Phalloidin (Alexa Flour 488) in the dark at room temperature for 1 h. After four washes with PBS, the coverslips were mounted with GelMount containing DAPI to label nuclei, and images were collected the following day. Fluorescence microscopy was performed using the Leica Confocal microscope (TCS, SP5 model). All parameters were kept constant (including exposure time) while acquiring images. Images were acquired in a 16-bit format and were analyzed using ImageJ software from single-plane images. Brightness and contrast adjustments were kept constant throughout the images. To measure podocyte recovery, actin reorganization was used as readout (49, 53). When recovery of the actin cytoskeleton as determined by the recurrence of stress fibers occurred in less than 20% of the area of the cell (at the point of greatest cell diameter), the podocyte was considered to be injured. When actin reorganization was seen in more than 20% of the area of the cell, the cells were scored as having recovered.

### Isolation of Neph1 complexes

GST-NEPH1-antibody and preimmunized serum affinity columns were prepared using N-hydroxysuccinimide (NHS)-activated Sepharose 4 Flow Fast (GE Healthcare) according to the manufacturer's instructions. The NEPH1-overexpressing podocyte lysate was loaded onto each column. Both Neph1-antibody and control columns were washed and eluted with glycine HCl buffer (pH 2.5). The eluate was neutralized with Tris (pH 8.0), and all eluates were later concentrated using a 10-kDa-cutoff centrifugal filter (Millipore). The concentrated protein from the NEPH1-antibody and control columns was separated by SDS-PAGE. Differentially visible bands were excised from the gel and submitted for tandem mass spectrometry (MS-MS) analysis at the Proteomics Core Facility of the University of Pennsylvania.

### Enzyme-linked immunosorbent assay

A competitive ELISA was performed as described previously with minor modifications (54). HGF (Sigma), 100 ng, was coated on individual wells of a 96-well Maxisorp Immunoplate (Nunc) and incubated at 4 °C overnight. The wells were blocked with 5% BSA (Sigma) in PBS for 4 h at 37 °C and then washed with 1× PBS, 0.1% Tween 20 solution (PBS-T). The wells in the plates were incubated with 200 ng of NEPH1, NEPHRIN, and MET proteins for 4 h at 37 °C alone and in combination with increasing concentrations (50, 100, and 150 ng) of HGF-binding NEPH1-peptide and NEPHRIN-Peptide 1, respectively. Following incubation, the wells were washed with PBS-T solution and incubated with NEPH1, NEPHRIN, and MET antibodies for 4 h. Post incubation, secondary antibody (HRP-conjugated) against the Fc region of human IgG1 mAbs at a dilution of 1:5000 in PBS containing 5% BSA was added and the plates were kept for 1 h at room temperature. The plates were then washed three times with PBS-T and twice with PBS and developed by adding 100 μl of substrate (3,3,5,5-tetramethylbenzidine) solution (Pierce). Incubation was done at room temperature, the reaction was stopped as the color developed by adding 100 μl of 2 M H_2_SO_4_, and absorbance at 450 nm was measured on a microplate reader (Biotek).

### shRNA-mediated knockdown of NEPH1 and SHP-2 in cultured podocytes

NEPH1 knockdown in cultured human podocytes was achieved using NEPH1-specific shRNA. The shRNAs in pLKO.1-puro (MET MISSION Lentiviral Transduction shRNA particle) were commercially purchased from Sigma (Cat. No. SHCLNV-NM_018240 and TRC number: TRCN0000147545 for NEPH1). SHP-2 knockdown in podocytes was generated as described (44). The transduction of the shRNA plasmids was performed according to the manufacturer's instructions. Selection for stably transduced cells was performed by culturing the cells in 2.5 μg/ml puromycin-containing medium, and knockdown was confirmed by Western blot.

### CRISPR-Cas9-based knock out of MET in cultured podocytes and HEK293 cells

MET knockout (KO) in cultured human podocytes and HEK293 cells was achieved using MET CRISPR-Cas9 KO and Met HDR plasmids according to the manufacturer’s instructions (55). The Met HDR plasmid was used for cotransfection with the Met CRISPR-Cas9 KO plasmid (h2) and designed for repair of the site-specific Cas9-induced DNA cleavage within the MET gene. During repair, the Met HDR plasmid incorporates a puromycin resistance gene to enable selection of stable KO cells and an RFP gene to visually confirm transfection. The plasmids were commercially obtained from Santa Cruz Biotechnology (Cat No. sc-400101-KO-2 and sc-400101-HDR-2). For complete allelic knockouts stable clones grown from single cells were isolated. The KO in the fully selected clones was confirmed by Western blotting using the MET-specific antibody.

### *Drosophila* nephrocytes as a model for HGF-induced renoprotection

The *Drosophila* experiments were designed and performed at the University of Maryland by Dr Wen and Dr Han. Briefly, *Drosophila* pericardial nephrocytes were dissected in artificial hemolymph and then treated under different conditions for 90 min at room temperature. The final concentration of PS was 1 mg/ml, HGF 50 ng/ml, NEPH1 peptide 250 μg/ml, NEPHRIN P1 25 μg/ml, NEPHRIN P2 25 μg/ml. The nephrocytes were then heat-fixed for 20 s and stained with an anti-SNS antibody from Britta George. For quantification, seven nephrocytes were analyzed from three flies for each treatment. These studies followed all the necessary regulations at the University of Maryland, although *Drosophila* studies do not require IRB approval at the University of Maryland.

### Statistical analysis

All statistical analyses were performed using GraphPad Prism 7 software. Each dataset is presented as mean ± SEM. The Mann–Whitney (nonparametric) test was performed to assess the statistical differences between two groups. To analyze differences between more than two groups, a one-way (Kruskal–Wallis test) or two-way (Sidak’s multiple comparison test) or Tukey's multiple comparisons test (one-way ANOVA) nonparametric analysis of variance was performed. Details of the statistical analyses used for each experiment are provided in the respective figure legends. A *p*-value of ≤0.05 was considered statistically significant.

## Data availability

The authors declare that all data supporting the finding of this study are available within this article and its supplementary information files or from the corresponding author (J. H. L.) upon request. The plasmids will also be available from the corresponding author (J. H. L.) upon request.

## Supporting information

This article contains supporting information (31).

## Conflict of interest

The authors declare that they have no conflicts of interest with the contents of this article.
