# Supplementary material for: Phosphorylation of slit diaphragm proteins NEPHRIN and NEPH1 upon binding of HGF promotes podocyte repair
Source: J Biol Chem. 2021 Aug 13;297(3):101079. doi: 10.1016/j.jbc.2021.101079 (PMC8429977; doi:10.1016/j.jbc.2021.101079)
Supplement: Supplemental Figures S1–S5 [file mmc1.pdf]

SUPPLEMENTARY DATA:

HGF-induced activation of NEPHRIN and NEPH1, a novel mechanism that participates in podocytes recovery from injury

Ashish K. Solanki[1], Ehtesham Arif[1], Pankaj Srivastava[2], Christopher M. Furcht[3], Bushra Rahman[1], Pei Wen[4], Avinash Singh[1], Lawrence B Holzman[5], Wayne R. Fitzgibbon[1], Milos N. Budisavljevic[1], Glenn P. Lobo[1], Sang-Ho Kwon[6], Zhe Han[4], Matthew J. Lazzara[7], Joshua H. Lipschutz[1,8*#], Deepak Nihalani[9*]

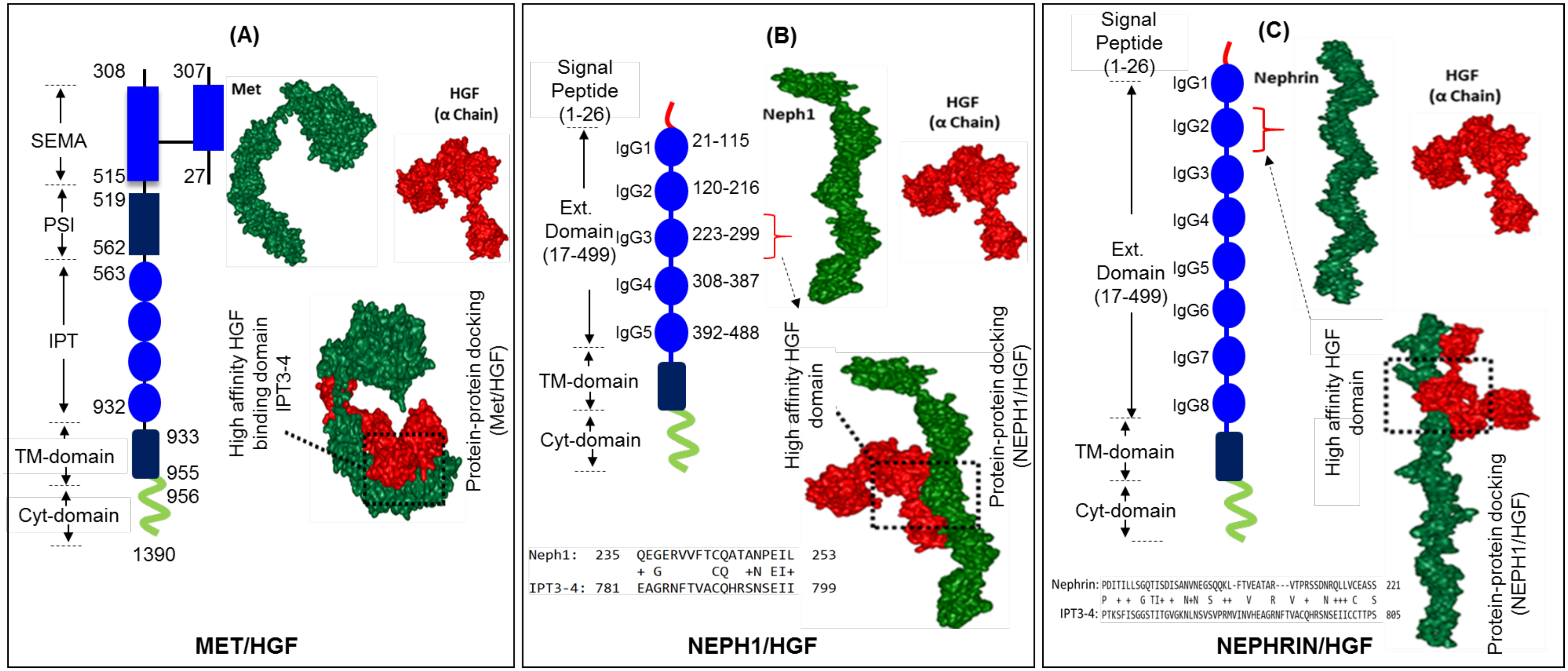

Supplementary Figure 1: Identification of putative HGF-interacting domains in NEPHRIN and NEPH1 through molecular modeling. (A-C) The high-affinity binding site of the MET receptor (IPT3-4 domain) with HGF was used as a template. The cartoon represents the respective domain architecture of MET (**A**), NEPH1 (**B**), and NEPHRIN (**C**). The alignment of protein sequences representing the extracellular domains of NEPH1 and NEPHRIN with IPT3-4 region of MET was performed using NCBI BLAST, which showed 32% and 23% identities with the IgG domain 3 of NEPH1 and IgG domain 2 of NEPHRIN, respectively. Peptides from these putative HGF binding sites were synthesized and used in binding and functional assays. Structural protein models for the HGF α chain, MET, NEPH1, and NEPHRIN extracellular domains were constructed using the protein fold recognition-based modeling server PHYRE2. Using Z dock, protein-protein docking with the HGF α chain was performed that identified the interacting regions.

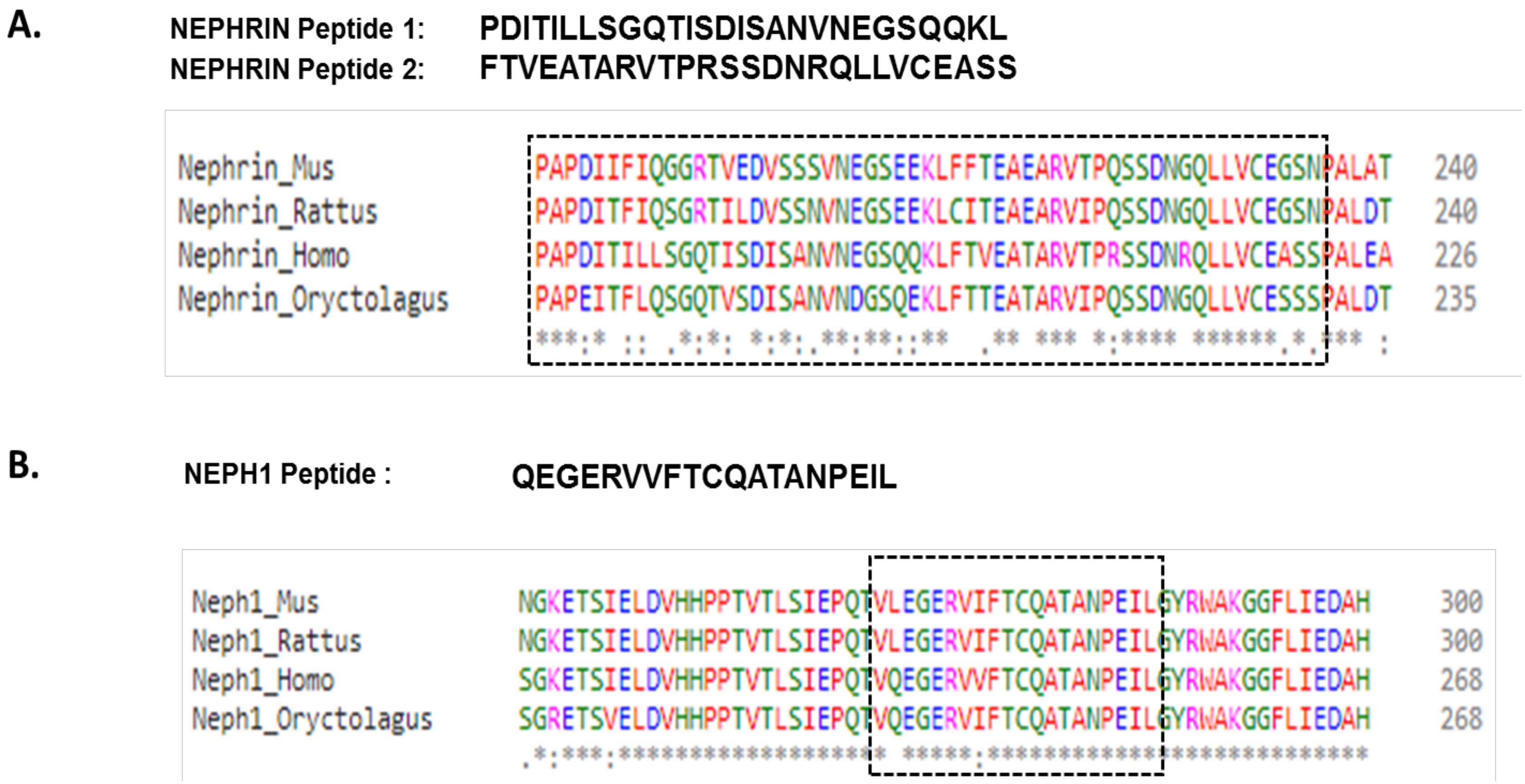

Supplementary Figure 2: The HGF-binding region in NEPHRIN and NEPH1 is highly conserved. Putative NEPHRIN (A) and NEPH1 (B) peptides that interact with HGF were highly conserved across multiple species including mouse, rat, human, and rabbit.

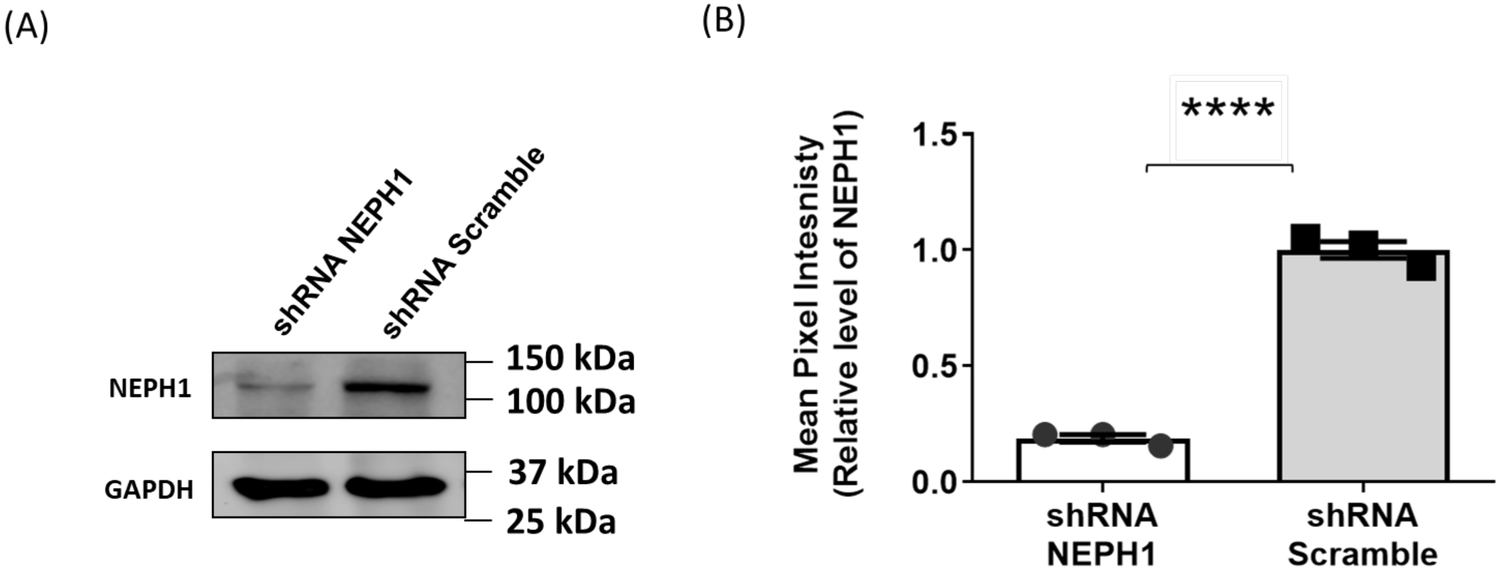

Supplementary Figure 3: NEPH1 knockdown in cultured human podocytes. (**A**) Podocytes with stable NEPH1 knockdown were generated using NEPH1-specific shRNA. NEPH1 knockdown was evaluated by western blotting with NEPH1 antibody. GAPDH was used to determine equivalent protein loading. (**B**) The relative decrease in NEPH1 levels were quantified. Data are presented as mean ± SEM, and p-values were calculated using a two-tailed student's t-test. **** = p≤ 0.0001. All experiments were performed in triplicate, repeated three times with similar results, and representative images of the results are presented in the figure.

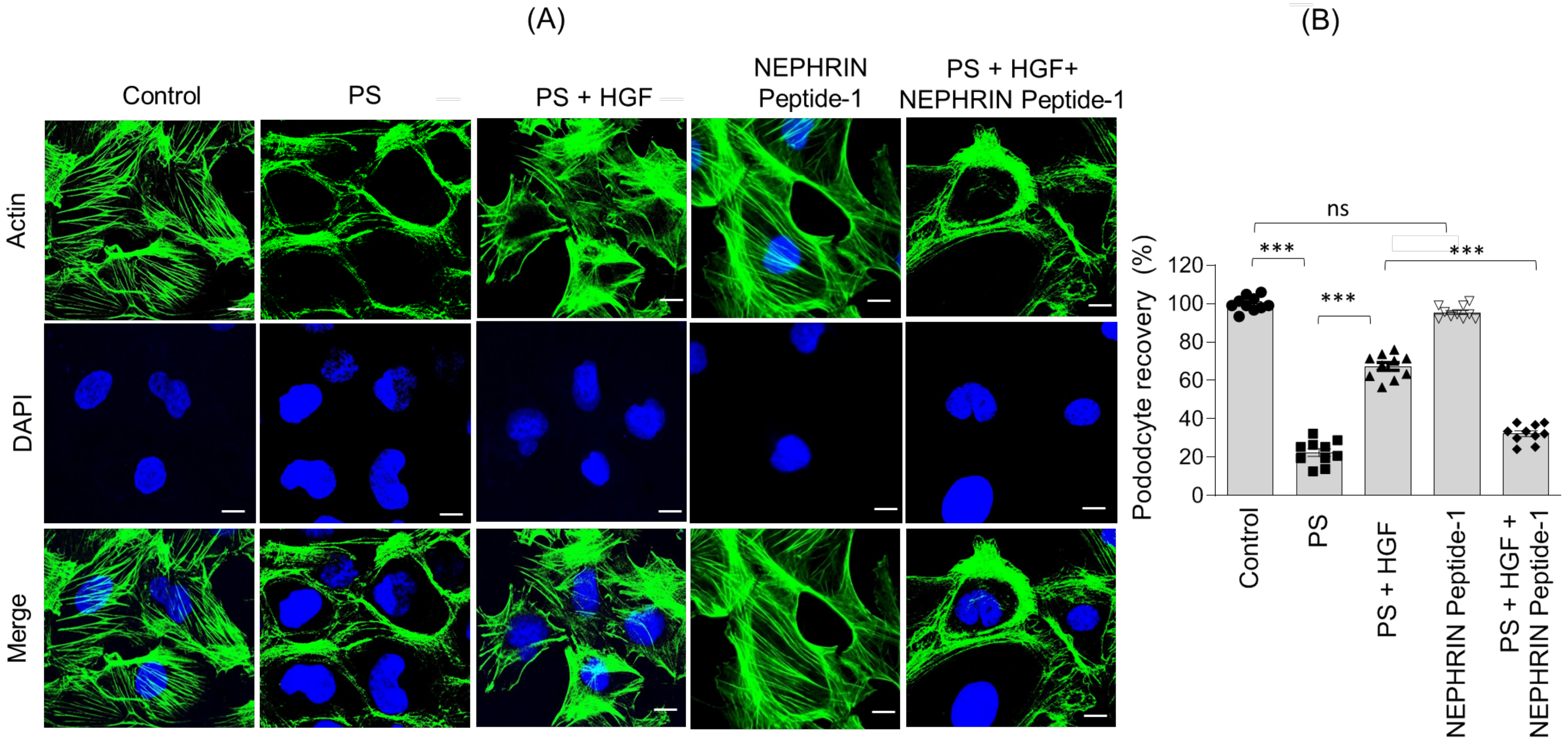

Supplementary Figure 4: HGF treatment repairs podocytes/nephrocytes in a NEPHRIN-dependent fashion: (**A & B**) Cultured human podocytes overexpressing NEPHRIN were treated with PS and actin cytoskeletal (green) disorganization was visualized by phalloidin staining. To induce recovery, HGF (50 ng/mL) was added to the PS-treated podocytes. The addition of NEPHRIN inhibitory peptide blocked HGF-induced recovery. 10 cells per experimental condition were evaluated from three experimental trials. Scale bar=25 µm. Data are presented as mean ± SEM, and p-values were calculated using the Tukey's multiple comparisons test (One-way ANOVA). ns= non-significant, *** = $p \leq 0.001$.

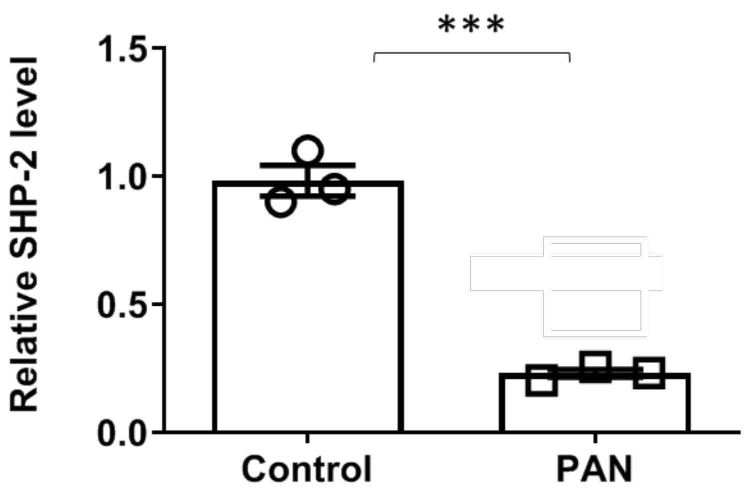

Supplementary Figure 5: Quantification of SHP-2 expression level in PAN-injured podocytes. The relative SHP-2 expression measured from the mRNA profiling data of podocytes injured with PAN shows down-regulation of SHP-2 expression (Gene Expression Omnibus accession number GSE124622, [31]). Data are presented as mean ± SEM, and p-values were calculated using a two-tailed Student's t-test. *** = p≤ 0.001. The experiment was performed in triplicate, repeated three times with similar results, and a representative image of the results is presented in the figure.
